# Berberine protects human renal proximal tubular cells from hypoxia/reoxygenation injury via inhibiting endoplasmic reticulum and mitochondrial stress pathways

**DOI:** 10.1186/1479-5876-11-24

**Published:** 2013-01-29

**Authors:** Wenli Yu, Mingwei Sheng, Rubin Xu, Jianjian Yu, Kang Cui, Jingkai Tong, Liying Shi, Hengchang Ren, Hongyin Du

**Affiliations:** 1 Department of Anesthesiology, Tianjin First Center Hospital, Tianjin, 300192, China; 2 Department of Immunology, Tianjin Medical University, Tianjin, 300070, China; 3 Department of Microbiology, Tianjin Medical University, Tianjin, 300070, China; 4 Department of Anesthesiology, Tianjin Chest Hospital, Tianjin, 300051, China; 5 Key Lab for Critical Care Medicine of the Ministry of Health, Tianjin, 300192, China

**Keywords:** Berberine, Kidney, Apoptosis, Endoplasmic reticulum stress, Mitochondria

## Abstract

**Background:**

Ischemia/reperfusion injury plays a crucial role in renal transplantation, and represents a significant risk factor for acute renal failure and delayed graft function. The pathophysiological contribution of endoplasmic reticulum and mitochondria stress to ischemia/reperfusion injury has also been highlighted. Berberine (BBR) has been showed to attenuate ischemia/reperfusion injury by inhibiting oxidative stress. The study was carried out to investigate whether the pretreatment of BBR could reduce hypoxia/reoxygenation (H/R)-induced injury by inhibiting mitochondria stress and endoplasmic reticulum stress pathways.

**Methods:**

The cultured human renal proximal tubular cell line HK-2 cells were exposed to 24 h hypoxia (5% CO_2_, 1% O_2_, 94% N_2_) followed by 3 h reoxygenation (5% CO_2_, 21% O_2_, 74% N_2_). And BBR was added to the culture medium 2h prior to the treatment. Then the cell viability, oxidative stress level, morphological change of apoptosis and apoptotic rate were determined. In addition, Western blot analysis was performed to identify the expression of apoptotic pathway parameters, including Bcl-2, Bax and cytochrome C involved in mitochondrial-dependent pathway and ER stress hallmarks such as glucose-regulated protein 78 and CCAAT/enhancer binding protein homologous protein.

**Results:**

H/R produced dramatic injuries in HK-2 cells. The cell viability and the oxidative stress level in group H/R was significantly decreased. The classical morphological change of apoptosis was found, while the apoptotic rate and the expression of proteins involved in mitochondrial stress and endoplasmic reticulum stress pathways increased (*p*<0.05). Administration of BBR significantly inhibited these H/R induced changes (*p*<0.05).

**Conclusion:**

This study revealed that BBR pretreatment serves a protective role against H/R induced apoptosis of human renal proximal tubular cells, and the mechanism is related to suppression of mitochondrial stress and endoplasmic reticulum stress pathways.

## Background

Ischemia/reperfusion injury (IRI), which occurs in 10%-20% of renal transplant (RTx) recipients [1], is a major cause of acute renal failure and increases the risk of delayed graft function and early mortality as well [2]. According to the reports [3,4], proximal tubular cells are the most sensitive renal cells to IRI and the main area of acute kidney injury. Therefore, protecting proximal tubular cells from IRI has been considered a reasonable strategy for significantly improving short- and long-term outcomes for RTx recipients and potentially expanding the use of “marginal donor organs” which are more susceptible to IRI [5].

Hypoxia during ischemia and subsequent reoxygenation upon reperfusion are thought to be the major culprits contributing to reactive oxygen species (ROS) production which leads to uncontrolled oxidative stress and the subsequent cell apoptosis [6,7]. During the injury, mitochondria and endoplasmic reticulum (ER) play important roles. Increasing evidence has demonstrated that oxidative stress interferes with not only redox-dependent reactions but also protein-folding capacity ultimately resulting in protein misfolding in the ER [8,9]. Related studies have been done in various cells such as neuronal-like rat pheochromocytoma cells [10], pancreatic β-cells [11] and so on [12], whereas no study has been carried out concerning their relationship in renal proximal tubular cell apoptosis induced by IRI.

Berberine (BBR) is an alkaloid extract from traditional medicine herbs. It has been used for long in oriental medicine to treat diarrhea [13], but not until recent decades have its benefits on anti-diabetic, anti-inflammatory and anti-tumor effects been realized. BBR is a novel glucose-lowering drug which acts by stimulating insulin secretion, sensitizing insulin activity and inducing glycolysis [14]. It also exerts anti-inflammatory effects by diminishing the expression of pro-inflammatory molecules including cyclooxygenase-2, interleukin-1 and tumor necrosis factor-α [15]. Its cytotoxic effects on most cancer cell types also highlight the promise of BBR as a potential multispectrum anticancer agent [16]. Additionally, investigations have shown that BBR can mitigate IRI of grafts by combating against oxidative stress [17]. However the molecular mechanisms of its protective effects are still not fully clarified.

Given the close association between mitochondria stress and ER stress, we can predict that BBR might protect against IRI by inhibition of apoptosis via mechanisms involving these two pathways. To test the assumption, the model in vitro of renal IRI by human proximal tubular cell line (HK-2) under hypoxia/reoxygenation(H/R) treatment was created. The goals of the present study were: (1) to confirm the existence of mitochondrial and ER stress pathways in H/R induced HK-2 cell apoptosis. (2) to show whether the renal protection conferred by BBR involves suppression of mitochondrial and ER stress in vitro.

## Methods

### Materials and reagents

The human renal proximal tubular cell line HK-2 was obtained from American Type Culture Collection (Manassas, VA, USA). Dulbecco Modified Eagle Medium (DMEM)/F12 and fetal bovine serum (FBS) were purchased from Gibco Technologies (Logan, UT, USA). BBR chloride was purchased from Sigma-Aldrich (St. Louis, MO, USA), and diluted in 10% dimethyl sulfoxide (DMSO) (Sigma, St.Louis, MO, USA) just before use (the final concentration of DMSO never exceeded 1% in both control and treated cells). Annexin V–fluorescein isothiocyanate (FITC) apoptosis detection kit was obtained from Biovision (Milpitas, CA, USA). The Hoechst staining kit was purchased from Beyotime (Jiangsu, China). The antibodies against Bax, Bcl-2, Cytochrome C, GRP78, CHOP, caspase-3, GAPDH and β-actin were purchased from Cell Signaling Technology (Beverly, MA, USA).

### Cell culture and H/R treatment

HK-2 cells were cultured in DMEM/F12 medium, supplemented with 10% heat-inactivated FBS. They were kept at 37°C in a 5% CO_2_ atmosphere. Before experimental intervention, confluent cultured cells were serum-starved for 24 h in DMEM/F12 supplemented with 0.1% FBS. Various concentrations of BBR were added 2 h before exposure to H/R. The groups were randomly divided into the following groups: (1) Control: cells were incubated in normoxic condition (5% CO_2_, 21% O_2_, and 74% N_2_) without BBR treatment. (2) BBR: cells were treated with BBR and incubated in normoxic conditions; (3) H/R: cells were exposed to 24 h of hypoxia (5% CO_2_, 1% O_2_, and 94% N_2_) followed by 12 h of reoxygenation (5% CO_2_, 21% O_2_, and 74% N_2_). (4) H/R+BBR: cells pretreated with BBR were exposed to 24 h of hypoxia followed by 3 h of reoxygenation.

### Cell viability assay

Cytotoxicity of BBR on HK-2 cells was tested by the a colorimetric assay using 3-(4,5)-dimethylthiahiazo (-z-yl)-3, 5-di-phenytetrazoliumromide (MTT) (Amresco, USA). Cells were seeded into 96-well plates (104 cells/well) and exposed to DMEM alone or treated with BBR (0, 10, 25, 50, 75, 100 μM) [18,19] for 24 h. Then the medium was removed and MTT dissolved in phosphate buffered saline (PBS) at a final concentration of 5 mg/mL was added to each well. After 4 h incubation, the culture was removed and cells were dissolved in DMSO. The absorbance was measured by an enzyme-linked immunosorbent assay (ELISA) analyzer (Thermo Fisher Scientific, Waltham, MA, USA) at a wavelength of 490 nm. Wells without cells were used as blanks. Results were expressed as percentages of control.

The optimal concentration of BBR exerting protective effects in H/R-induced HK-2 cells was also determined by the MTT assay. The following formula was used to calculate cell viability.

$$\begin{array}{c} \text{Viability}\left(\%\right)=\left({\text{OD}}_{490,\text{sample}},–,{\text{OD}}_{490,\text{blank}}\right)\;⁄\\ \;\left({\text{OD}}_{490,\text{control}},–,{\text{OD}}_{490,\text{blank}}\right)\times 100 \end{array}$$

### Detection of malondialdehyde (MDA) and superoxide dismutase (SOD)

Levels of MDA and SOD in culture medium were detected by thiobarbituric acid (TBA) colorimetric method using commercially available kits which were purchased from the Jiancheng Bioengineering Institut (Nanjing, China). All procedures were performed according to the manufacturers` recommendation in the kit manuals.

### Morphological detection and quantification of apoptosis

Apoptosis was determined morphologically by inverted microscope (Eclipse TS100, Nikon, Japan) and Hoechst33258 staining. As for Hoechst33258 staining, HK-2 cells were grown in 6-well plates and treated as described above. Then they were washed with PBS and stained with Hoechst 33258 for 5 min. The nuclear morphology of cells was visualized by fluorescence microscopy (Eclipse TE300, Nikon, Japan) at 460 nm. Cells that had chromatin condensation and nuclear fragmentation were apoptotic cells [20].

Quantification of apoptotic cells was determined by Annexin V-FITC/PI staining. The adherent cells were collected by centrifugation and then resuspended in 500 μl 1× binding buffer. Then they were stained with 5 μL Annexin V–FITC and 5 μl propidium iodide (PI) (50 μg/mL) and incubated at room temperature for 5 min in the dark. The cells were analyzed by a flow cytometry (Becton Dickinson FACS Vantage SE, Sanjose, USA). The results were shown as quadrant dot plots with intact cells (Annextin V-/PI-), early apoptotic cells (Annextin V+/PI-), late apoptotic cells(Annextin V+/PI+) and necrotic cells (Annextin V-/PI+). The number of each kind of cells was expressed as percentages of the number of total stained cells.

### Western blot analysis

HK-2 cells were washed twice with cold PBS and harvested. Total cell extracts were lysated in the buffer containing complete protease inhibitors cocktail. Cell lysates were centrifuged at 15, 000×g for 30 min at 4°C and then supernatants were collected for western blot analysis. Equal amounts of samples were electrophoresed in 12% SDS-polyacrylamide gel electrophoresis (PAGE) and transferred onto polyvinylidene difluoride membranes. Nonspecific binding would be blocked with 5% dried skim milk in Tris-buffered saline and incubated with specific primary antibodies at 4°C for 12 h. Following three times washes with Tris-buffered saline, the bound primary antibodies were detected by appropriate horseradish peroxidase-conjugated secondary antibodies (diluted 1:5000). The blotted membranes were detected by enhanced chemiluminescence (ECL) reagents (American Bioscience, USA) and autoradiography. Data analysis was finished by Image J, measuring the densities of immunoreactive bands.

The analysis was performed using primary antibodies against glucose-regulated protein 78 /immunoglobulin heavy chain- binding protein (GRP78 /Bip), caspase-3, Bax, Bcl-2, cytochrome C and CHOP at the optimal dilution. GAPDH or β-actin was used as an internal control.

### Statistical analysis

All values were expressed as mean ± S.D. (Standard Deviation). Statistical differences were performed by one-way analysis of variance (ANOVA). The level of statistical significance was defined as *P* <0.05. The SPSS 13.0 (Chicago, IL, USA) statistical software package was used for all calculations.

## Results

### BBR improved the cell viability of H/R-induced HK-2 cells

**Figure 1:**
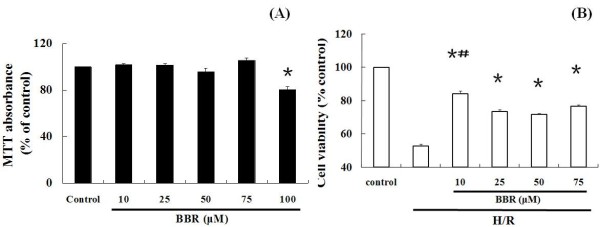
**Effect of BBR on cell viability in HK-2 cells by MTT assay.** (**A**) Effect of BBR on HK-2 cells under normoxic condition by MTT assay. The results were expressed as mean ±S.D. (n=5). ^*^p<0.05 vs. control. (**B**) Effect of BBR on H/R induced HK-2 cells by MTT assay. The results were expressed as mean ±S.D. (n=5). ^*^p<0.05 vs. H/R group without treatment; ^**#**^*p*<0.05 vs. other H/R + BBR groups.

As showed in Figure 1A, under normoxic condition, the doses of BBR below 75 μM caused little effects on cell proliferation. However BBR significantly enhanced cytotoxicity with the dose of 100 μM (*P*<0.05).

After HK-2 cells were pre-treated with various doses of BBR (except 100 μM) and exposed to H/R, we measured cell viability by MTT assay again. As showed in Figure 1B, compared with control, the cell proliferation was markedly inhibited after H/R treatment (*P*<0.05), which was significantly improved by BBR. Among all of the concentrations, 10 μM BBR represented the optimal effect, improving cell viability by approximately 85% *P*<0.05). Based on this result, all subsequent experiments were performed with 10 μM BBR.

### BBR reduced the oxidative stress of H/R-induced HK-2 cells

**Figure 2:**
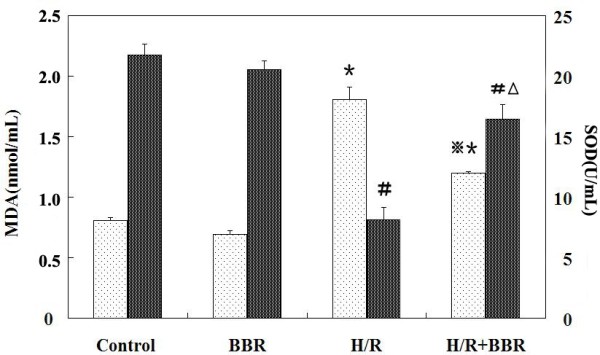
**MDA content (white bars) and SOD activity (black bars) in the culture medium.** Experiments were performed at least three times with similar results. The results were expressed as mean ±S.D. (n=5). ^*^p<0.05 vs. control, ^**#**^*p*<0.05 vs. control, ^※^*p*<0.05 vs. H/R group, ^△ ^*p*<0.05 vs. H/R group.

Figure 2 shows the effect of BBR on MDA content and SOD activity in cultured lipid of HK-2 cells. In comparison with Control group (SOD: 21.75±0.09 U/mL; MDA: 0.81±0.02 nmol/mL), H/R treatment decreased SOD activity (p<0.05) whereas an increase in MDA content (p<0.05) occurred. BBR treatment effectively blocked the increase of MDA with an elevation of SOD activity, exhibiting significant anti-oxidative activity (SOD: 16.46±1.18 U/mL; MDA:1.20±0.11 nmol/mL) (*P*<0.05).

### BBR protected against H/R-induced apoptosis in HK-2 cells

**Figure 3:**
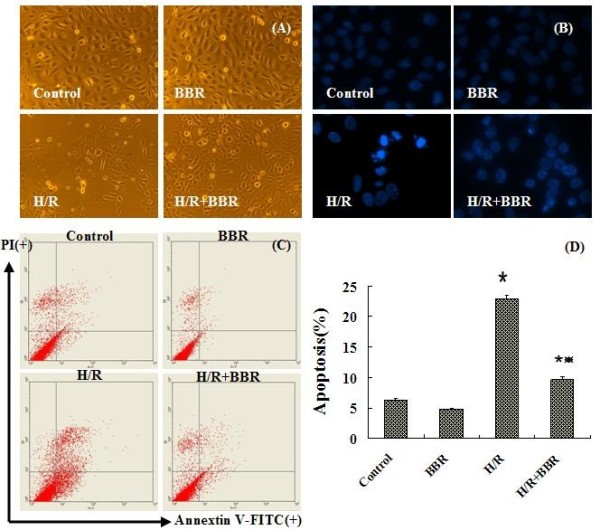
**Effect of BBR on H/R-induced apoptosis in HK-2 cells.** (**A**) Under the inverted phase contrast microscope (×100), control and BBR groups showed the normal elliptical morphology. H/R caused extensive blebbing morphology and a decreased cell population, while BBR prevented the injury. (**B**) Under fluorescence microscope (×400), apoptotic cells stained with Hechst33258 showed nucleic fragmentation with dense chromatin. They were the typical morpyological changes of apoptosis. (**C**) Quantitative assessment of apoptotic cells by annexin V-FITC/PI staining. Intact cells were V-/PI-, early apoptotic cells were V+/PI- , late apoptotic cells were V+/PI+ and necrotic cells were V-/PI+. (**D**) Flow cytometry results were showed as quantitative bar graphs. The results were expressed as mean ±S.D. (n=5). ^*^p<0.05 vs. control, ^**#**^*p*<0.05 vs. H/R group without BBR treatment.

Figure 3A shows the cellular morphology of HK-2 cells exposed to normoxia, BBR, H/R or H/R combined with BBR under an inverted phase contrast microscope (×100). Cells grown under normoxia or BBR had normal elliptical morphology, whereas H/R induced cells appeared with extensive blebbing and a decrease in population. Compared to the H/R group, BBR blocked apoptosis in an increased cell population with slighter blebbing.

To further elucidate the effect of BBR on apoptosis, Hoechst33258 staining was assessed (Figure 3B). Under fluorescent microscope (×400), the majority of HK-2 cells in control and BBR groups showed normal morphology with round regular nuclei. In contrast, apoptotic bodies were seen in H/R induced cells. However the pretreatment of BBR effectively reduced HK-2 cells apoptosis according to the restored morphology.

Annexin-V FITC/PI staining confirmed the anti-apoptotic effects of BBR quantitatively (Figures 3C, D). Compared with control group, the portion of annexin-V(+)/PI(−) cells in H/R group increased from 6.25% to 22.95% (*P*<0.05). However pretreatment with BBR 2 h prior to H/R significantly attenuated the percentage of annexin-V(+)/PI(−) cells to 9.59% (*P*<0.05), demonstrating the anti-apoptotic effect of BBR.

### BBR inhibited caspase-3 activation in H/R induced HK-2 cells

**Figure 4:**
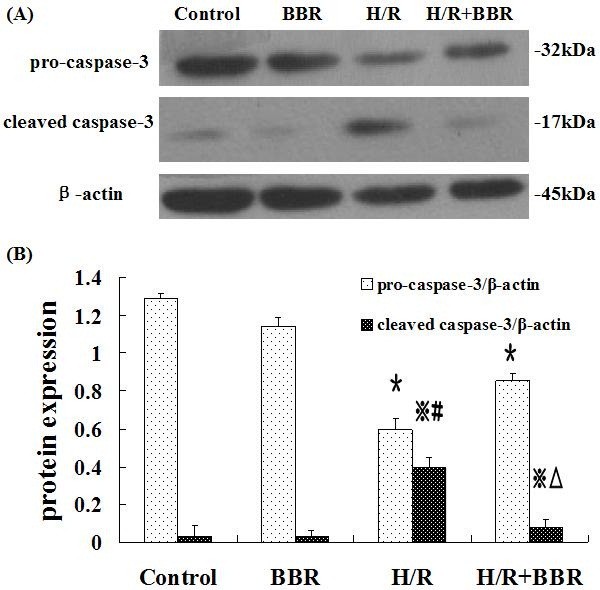
**Effects of BBR on expression of pro-caspase-3 and cleaved caspsae-3.** The results were expressed as mean ±S.D. (n=5). ^*^p<0.05 vs. control, ^**#**^*p*<0.05 vs. control, ^※^*p*<0.05 vs. H/R group, ^△ ^*p*<0.05 vs. the group with only H/R treatment.

Another important piece of evidence to confirm the anti-apoptotic effect of BBR was from the detection of caspase-3 by western blot analysis. Activation of caspase-3 from pro-caspase-3 is a key downstream event involved in the initiation and execution of apoptosis. To confirm whether caspase-3 was involved in the H/R-induced apoptosis and the effect of BBR on the apoptosis, we examined the expression of procaspase-3 and cleaved caspase-3 by western blot analysis (Figure 4). Compared with the control group, procaspase-3 was down-regulated in the H/R induced HK-2 cells, whereas the up-regulated expression level of cleaved caspase-3 revealing apoptosis was detected in H/R induced cells. And pretreatment with BBR antagonized this effect by inhibiting caspase-dependent pathway.

### BBR attenuated the apoptosis through mitochondrial-dependent pathway in HK-2 cells

**Figure 5:**
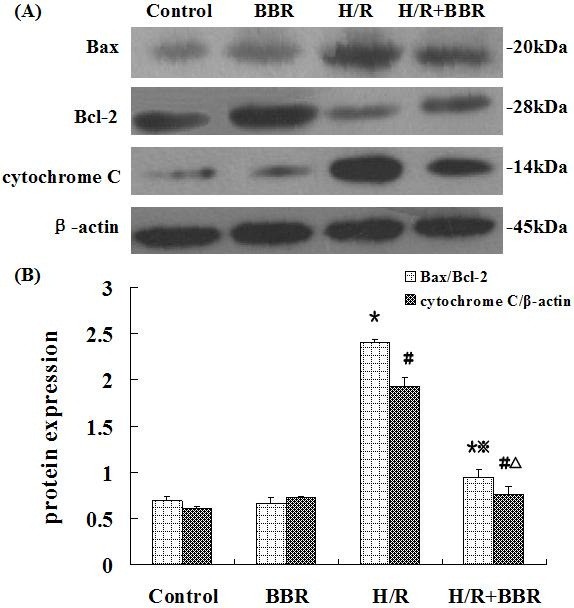
**Effects of BBR on mitochondria stress markers in HK-2 cells.** The results were expressed as mean ±S.D. (n=5). ^*^p<0.05 vs. control, ^**#**^*p*<0.05 vs. control, ^※^*p*<0.05 vs. H/R group, ^△ ^*p*<0.05 vs. the group with only H/R treatment.

It’s known that the mitochondria-dependent pathway of apoptosis is regulated by Bcl-2 family members, such as the anti-apoptotic protein Bcl-2, pro-apoptotic protein Bax which are critical for releasing cytochrome C and the following downstream caspase activation. To investigate whether mitochondria were responsible for executing H/R induced apoptosis, we analyzed the expression of Bcl-2, Bax and cytochrome C (Figure 5). With H/R treatment, the protein level of Bax and cytochrome C were upregulated while the level of Bcl-2 was downregulated. As shown in Figure 5B, the ratio of Bax/Bcl-2 increased by 3.4 fold under H/R treatment, compared with control group. After BBR treatment, the ratio was reduced and the expression of cytochrome C decreased at the same time, indicating that BBR can attenuate apoptosis through mitochondrial-related pathway in HK-2 cells.

### BBR attenuated the apoptosis through ER-dependent pathway in HK-2 cells

**Figure 6:**
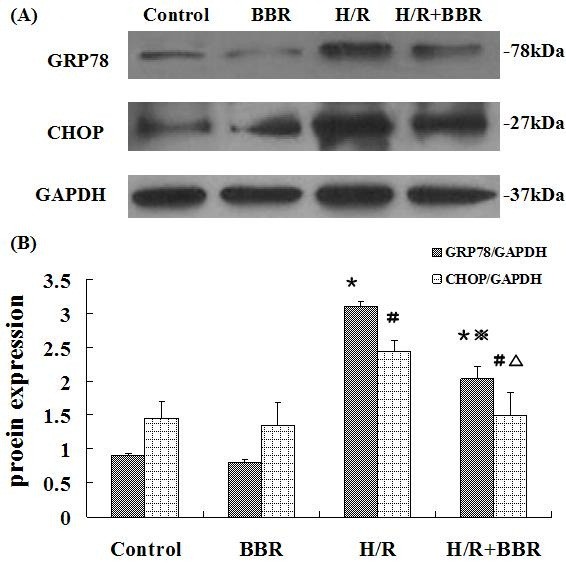
**Effects of BBR on ER stress markers in HK-2 cells.** The results were expressed as mean ±S.D. (n=5). ^*^p<0.05 vs. control, ^**#**^*p*<0.05 vs. control, ^※^*p*<0.05 vs. H/R group, ^△ ^*p*<0.05 vs. the group with only H/R treatment.

In addition to the mitochondrial-related pathway, ER stress was also evaluated on H/R induced apoptosis. At this point, two ER stress hallmarks GRP78 and CHOP were evaluated. As shown in Figure 6, GRP78 and CHOP protein levels increased after H/R and were suppressed by BBR treatment, indicating that BBR pre-treatment could ameliorate H/R-induced ER stress in HK-2 cells.

## Discussion

For the first time, we provide in vitro evidence of potential therapeutic value of BBR in renal IRI via inhibiting ER stress and mitochondrial stress pathways. Results showed that pretreatment with BBR inhibited oxidative stress and subsequent apoptosis in HK-2 cells subjected to H/R injury. Mechanisms underlying the protective effects may be largely attributed to the deactivation of ER stress and mitochondrial stress pathways.

It is believed that in the renal IRI, oxidative stress caused by ROS is one of the most critical mechanisms involved in the acceleration of cellular damage and thus extension of apoptosis and renal dysfunction [21]. Among biomarkers of oxidative stress, malondialdehyde (MDA) and superoxide dismutase (SOD) are known as two sensitive indicators [22]. MDA is the end product of lipid peroxidation [23]. These are considered to be indicators of oxidative stress as well as lipid peroxidation. SOD is an oxygen radical scavenger, which converts superoxide anion radicals to the upper stream of reactive oxygen metabolism cascade and protects cells against damage [24]. Relatively low content of antioxidant enzymes in the kidney makes it more vulnerable to the oxidative stress [25]. In the present study, increased level of MDA and decreased activity of SOD were noticed in the H/R induced HK-2 cells. However BBR restored the SOD activity to nearly control level. Compared with H/R group, MDA level was significantly reduced by BBR administration. Our study demonstrates that BBR treatment significantly alleviates oxidative stress by reducing renal MDA levels and increasing SOD activity of HK-2 cells following H/R injury.

Oxidative stress can contribute to apoptosis which has important role in the pathogenesis of renal IRI. The anti-apoptotic effect of BBR on H/R induced HK-2 cells was therefore tested. Hoechst33258 staining, annextin V-FITC/PI flow cytometry and the expression of caspase-3 were employed as apoptotic biomarkers. Under inverted contrast microscope, the obvious apoptotic morphological changes with extensive blebbing and a decreased cell population in H/R induced HK-2 cells were observed. Hoechst33258 staining showed nuclei fragmentation represented as condensed chromatin and bright staining. This apoptotic effect was further validated by the significant increase of the apoptotic cell population by flow cytometry. Berberine pretreatment significantly inhibited these H/R induced changes above. The expression of apoptotic proteins cysteinyl aspartate-specific protease-3 (caspase-3) including pro-caspase-3 and cleaved caspase-3 was examined. It is known that the observation of the cleavage of pro-caspase-3 reveals the activation of apoptosis. In this part, the down-regulation of pro-caspase-3 and the up-regulation of cleaved caspase-3 were found at the same time. Compared with the H/R group without BBR treatment, BBR reduced the expression of apoptotic proteins towards control levels.

The signal pathways through which BBR exerted its protective effects were investigated. One of the apoptotic mechanisms involved was via a mitochondrial stress pathway. The Bax/Bcl-2 ratio is a parameter for description of apoptotic state [26]. Increase of this ratio will alter the mitochondrial membrane permeability, leading to the release of cytochrome C. Then the downstream effector caspase-3 is activated, following with chromatin condensation and DNA fragmentation [27]. In this study, the increase of Bax/Bcl-2 ratio and cytochrome C in H/R induced HK-2 cells indicated that the cells had undergone apoptosis by a mitochondrial-dependent pathway, while these parameters decreased in the group pretreated with BBR. It appears that the ability of BBR to attenuate oxidative stress is partly by inhibiting the mitochondrial-related apoptosis.

ER stress has been implicated in the development of apoptosis [28,29]. ER is the site of protein synthesis, folding and maturation [30]. The perturbation of ER homeostasis can result in the accumulation of unfolded or misfolded protein, collectively called ER stress [31]. Under the pathological state, ER-localized chaperones including glucose-regulated protein 78 (GRP78) are induced and then a protein degrading system is initiated. The pro-apoptotic transcription factor C/EBP homologous protein 10 (CHOP) is also known as growth-arrest and DNA-damage inducible gene 153 (GADD153). This protein is a member of C/EBD transcription factor family that heterodimerizes with other C/EBDs and can be induced by ATF4. With the activation of those proteins, apoptosis takes place [32]. The western blot analysis revealed that exposure to H/R in HK-2 cells increased the expression of GRP78 and CHOP, indicating that ER stress participated in H/R induced apoptosis. With the treatment of BBR prior to H/R injury, those parameters were significantly reduced.

**Figure 7:**
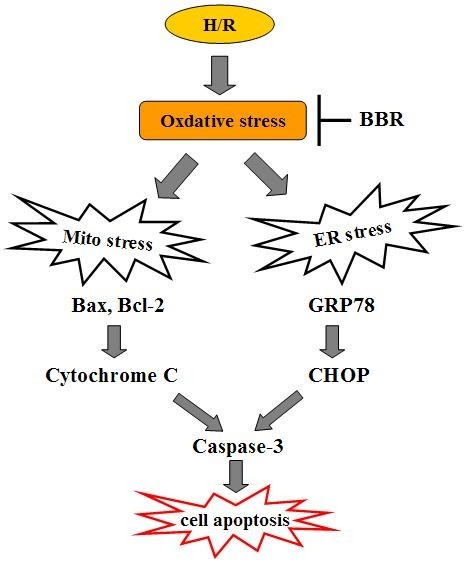
**Schematic diagram of the signal pathways involved in H/R-induced apoptosis in HK-2 cells.** Proposed models represent that H/R induces HK-2 cell apoptosis through oxidative stress-regulated mitochondria-dependent and ER stress-triggered signaling cascades.

A schematic representation of how BBR protects HK-2 cells against H/R induced apoptosis is shown in Figure 7. The H/R model is exposed to an oxidative stress. This stimulates the activation of both mitochondrial stress and ER stress pathways. With mitochondrial stress, the expression of Bax and Bcl-2 will be altered resulting in cell apoptosis. Cytochrome C is then released from mitochondria and the apoptotic effector protein caspase-3 is activated. Similarly, oxidative stress also activates the ER stress pathway. It causes the release of the GRP78 and CHOP and subsequent apoptosis. Both mitochondrial stress and ER stress orchestrate the apoptosis in H/R induced HK-2 cells. However, pretreatment with BBR significantly increased the percentage of viable HK-2 cells through inhibiting ER stress and mitochondrial-dependent pathways. The molecular mechanism of BBR's anti-apoptotic effect against H/R injury was reported in human renal tubular epithelial cells for the first time. Similar study was just found in pig renal tubular epithelial cells that the treatment with BBR exerted a protective effect against peroxynitrite-induced damage. A potential explanation for this was the reduction of cellular ONOO^-^ generation and DNA fragmentation [33]. Besides renal tubular epithelial cells, BBR was also effective against H/R induced cardiomyocytes or glial cells. In rat cerebral IRI model [34], BBR administration mitigated apoptotic process by inhibiting the expression of BACE via activation of the ERK1/2 pathway. Apoptosis of cardiomyocytes could be inhibited by BBR via protecting mitochondria, inhibiting an increase in the AMP/ATP ratio and AMPKa phosphorylation as well as elevating Bcl-2 expression [35]. A growing body of reports supports the evidence that BBR has pro-apoptotic potential via several different mechanisms. In human glioblastoma T98G cells, apoptosis was mediated by BBR through ER stress accompanying mitochondrial dysfunction [36]. The same effect was found in breast cancer cells [37] and oral cancer cells [38], in which BBR inhibited the transcription factor AP-1 expression. We thus propose that BBR might have multiple cellular targets and distinct effects that may be responding cell type specific. To better understand the molecular events regulating cell death after BBR treatment, numerous cellular proteins on the network of apoptosis need to be identified.

It should be pointed out that there are some limitations in our study. First of all, our in vitro experiments tested the effect of BBR on H/R induced apoptotic protein markers expression. But the downstream pathway and the cross-talk between ER stress and mitochondrial stress have not been investigated. Vance [39] has found that mitochondria and ER form close contacts with mitochondrial-associated membranes which have pivotal roles in numerous functions including Ca^2+^ signaling, energy metabolism, apoptosis and so on. The signal communication between the ER and mitochondria in the protective effect of BBR is still a fascinating emerging area of investigation. What’s more, the dosage-dependent effects and mechanisms involved are still not clear. In our study, the viability of BBR in H/R induced HK-2 cells was first studied by MTT assay. The treatment with 10, 25, 50, 75 μM BBR in H/R induced HK-2 cells enhanced cell viability in a dose-dependent manner and 10 μM was the most effective dose. Considering that MTT assay is a primary screening test which does not distinguish apoptosis and necrosis, further work must be carried out to clarify the specific effect of BBR. Lastly, our experiment is limited in vitro. In vivo experiments are required to gain a coherent understanding of mitochondria-ER nexus under the more complex internal environment. Additional research in animal models of renal IRI are in progress in our laboratory.

## Conclusions

The present study provides robust in vitro evidence that BBR can prevent H/R-induced oxidative stress and apoptosis. The mechanisms involved can be attributed to the inhibition of both ER and mitochondrial-dependent pathways. Our results may give evidence that BBR, a traditional herbal medicine, has potential to be a suitable candidate for preventing renal IRI by targeting mitochondria and ER. Before the clinical therapeutic application of this agent, further investigations should be performed.

## Abbreviations

IRI: Ischemia/reperfusion injury; RT: Renal transplant; ER: Endoplasmic reticulum; BBR: Berberine; H/R: Hypoxia/reoxygenation; MDA: Malondialdehyde; SOD: Superoxide dismutase; PAGE: SDS-polyacrylamide gel electrophoresis; CHOP: C/EBP homologous protein 10; GADD153: Growth-arrest and DNA-damage inducible gene 153; ROS: Reactive oxygen species; Caspase-3: Cysteinyl aspartate-specific protease-3; ECL: Enhanced chemiluminescence; DMSO: Dimethyl sulfoxide; MTT: 3-(4,5)-dimethylthiahiazo(−z-yl)-3,5-di-phenytetrazoliumromide.

## Competing interests

The authors declare that they have no competing interests.

## Authors’ contributions

HD and WY drafted the manuscript, designed experiments, analyzed and interpreted data and performed statistical analysis. MS contributed to the design of the study, discussed the data and helped to draft the manuscript. JY and KC carried out cytotoxicity experiments and western blot analysis. RX and HR conducted the flow cytometry analysis. All authors read and approved the final manuscript.
